# Climigration? Population and climate change in Arctic Alaska

**DOI:** 10.1007/s11111-016-0259-6

**Published:** 2016-06-23

**Authors:** Lawrence C. Hamilton, Kei Saito, Philip A. Loring, Richard B. Lammers, Henry P. Huntington

**Affiliations:** 1 Sociology Department, University of New Hampshire, Durham, NH 03824 USA; 2 School of Environment and Sustainability, University of Saskatchewan, Saskatoon, SK S7N 5C8 Canada; 3 Water Systems Analysis Group, Institute for the Study of Earth, Oceans, and Space, University of New Hampshire, Durham, NH 03824 USA; 4 Huntington Consulting, 23834 The Clearing Dr., Eagle River, AK 99577 USA

**Keywords:** Climate, Migration, Climigration, Alaska, Arctic, Erosion

## Abstract

Residents of towns and villages in Arctic Alaska live on “the front line of climate change.” Some communities face immediate threats from erosion and flooding associated with thawing permafrost, increasing river flows, and reduced sea ice protection of shorelines. The term *climigration*, referring to migration caused by climate change, originally was coined for these places. Although initial applications emphasized the need for government relocation policies, it has elsewhere been applied more broadly to encompass unplanned migration as well. Some historical movements have been attributed to climate change, but closer study tends to find multiple causes, making it difficult to quantify the climate contribution. Clearer attribution might come from comparisons of migration rates among places that are similar in most respects, apart from known climatic impacts. We apply this approach using annual 1990–2014 time series on 43 Arctic Alaska towns and villages. Within-community time plots show no indication of enhanced out-migration from the most at-risk communities. More formally, there is no significant difference between net migration rates of at-risk and other places, testing several alternative classifications. Although climigration is not detectable to date, growing risks make either planned or unplanned movements unavoidable in the near future.

## Introduction

The term *climigration* was coined by Alaska human rights lawyer Robin Bronen to describe the “forced permanent migration of communities due to climate change” (Bronen 2009:68). Her paradigmatic examples are remote Alaska villages such as Kivalina, Newtok, Shaktoolik, and Shishmaref, where predominantly indigenous populations face growing threats to infrastructure and safety from climate-linked erosion and flooding. Thawing permafrost, reduced shore protection from sea ice, and increasing river flows are consequences of Arctic climate change that impact these communities (Overpeck et al. 2005; NOAA 2015). Relocation seems inevitable given their geographical vulnerability, but that is not happening so far because of social resistance, difficulty, and high costs (GAO 2009; Huntington et al. 2012). New governance and institutional strategies are needed to deal with this new category of displacement (Bronen and Chapin 2013; Marino 2012, 2015). Apart from the personal, family, and cultural dislocations of moving, funding also remains a large obstacle: A 2006 study estimated relocation costs for Kivalina alone (population below 400 at the time) as $155–$251 million, depending on the site chosen (USACE 2006). Much of that cost would go toward site work and airport construction, new buildings, water/sewer system, and landfill. Site choice itself remains problematic. A proposed alternative location favored in a poll of village residents (although with some dissent) was described as “geotechnically inappropriate” by USACE because it sits on permafrost, or soils mostly frozen year-round. Moreover, this alternative location is vulnerable to future threats from coastal erosion as well. Newtok, Shishmaref, and other places face similar costs and complications for relocation (Tribal Climate Change Project 2010).

Rural Alaska communities commonly have birth rates well above replacement levels (Hamilton and Mitiguy 2009). The population growth that would otherwise result, however, can be significantly augmented, offset or even reversed by migration, which fluctuates widely in some of these small places (Hamilton and Seyfrit 1994a, b; Hamilton 2010). Out-migration reflects a variety of push and pull factors, including economic, educational, or social opportunities elsewhere. Movers often are people with greater human and social capital, and include more adult women than men. Such departures, not balanced by inflow of people with comparable attributes, have been identified as a threat to the viability of rural communities in Alaska (Martin et al. 2008; Gerlach et al. 2011) and elsewhere (Corbett 2005, 2007).

Might some rural community out-migration in Alaska already be climigration, happening despite government inaction but through human agency and indirect drivers (Black et al. 2011)? To answer this question, we look to a recently updated demographic database on Arctic Alaska that covers many of the places most threatened by climate-linked erosion (Saito et al. 2015). The database, containing annual time series of population and net migration from 43 communities over 1990–2014, provides an opportunity to systematically test for climigration by comparing out-migration rates from the most threatened communities with those from other, generally similar places.

## Climate and migration

Historical and contemporary observations establish that environmental factors can influence migration decisions, particularly among the most vulnerable (Hunter 2005). Geographic vulnerability by itself could be comparatively straightforward, as with decadal-scale projections of sea-level rise along coastlines (Curtis and Schneider 2011). Behavioral response to environmental pressures, however, tends to be socially mediated and complex, as seen even in modeling studies that take social factors into account (Entwisle et al. 2016). Environmental “push” forces are clearest in the wake of disasters, including climate-related disasters such as Cyclone Aila in Bangladesh (Islam and Hasan 2015) or Hurricane Katrina in New Orleans (DeWaard et al. 2016; Finch et al. 2010), but these affect people with diverse resources quite differently.

Slower climate-linked disasters such as crop failures from drought have been implicated among the causes of violent conflicts, which become an impetus for migration as well (Parenti 2011). Such effects could drive out-migration and destabilize weak states, making climate change a “threat multiplier” for Europe (Sabathil 2010:65). Authors of the Intergovernmental Panel on Climate Change (IPCC) Fifth Assessment Report agreed on the seriousness of displacement threats, while expressing low confidence in specific quantitative projections due to the complex, multi-causal nature of migration (Field et al. 2014:20).

Migration’s complex, multi-causal nature has been highlighted in social science research. Seemingly direct environmental influences are filtered by social factors including differences in social position, family resources, adaptation options, or household strategies to diversify risks (Hunter et al. 2015). Qualitative case study and ethnographic research portrays social complexity at “ground level;” other studies applying event history analysis to individual or household-level data take a nuanced quantitative approach. For example, temporary and permanent migration responding to rainfall conditions in Burkina Faso has been analyzed by Henry et al. (2004). Loebach (2016) examines the migration response in Nicaragua to Hurricane Mitch. Massey et al. (2010) estimate relationships between out-migration and environmental pressures such as firewood scarcity, declining agricultural productivity, and declining land cover. In agreement with qualitative research, event history analyses find variation in the duration and distance of moves, partly influenced by individual, family, and social factors.

Other methods converge on this conclusion that climate effects tend to be contingent. In a study of displacement caused by riverbank erosion in Bangladesh, Hutton and Haque (2004) highlight social differences in vulnerability. Comparing the Arctic Alaska village of Shishmaref with Nanumea, a low-lying atoll in the Polynesian island nation of Tuvalu, Marino and Lazrus (2015) observe that both communities face imminent, existential dangers associated with climate change. Their respective populations (around 500–600 people each) have gained international prominence as potential “climate refugees.” Even in these extreme cases, however, the authors note that disaster risks interact with other migration pressures, which influence individual, family, and community migration decisions in complex ways.

Given the entanglement among possible motivations to leave or stay, detecting specifically climatic effects on migration remains a challenge. In this paper, we take a novel quasi-experimental approach using annual population and migration time series on 43 Arctic Alaska towns and villages, some of which face immediate, recognized threats related to climate. The data support two kinds of analysis that could isolate climatic from other migration effects: tracking temporal change in migration rates within each community, or comparing median migration rates between otherwise generally similar groups of threatened and non-threatened communities. A temporal surge in migration as climate risks emerged, or significant contrasts between threatened and non-threatened places, could give evidence for the existence and scale of climate-induced migration.

## Alaska’s climate-threatened communities

**Fig. 1:**
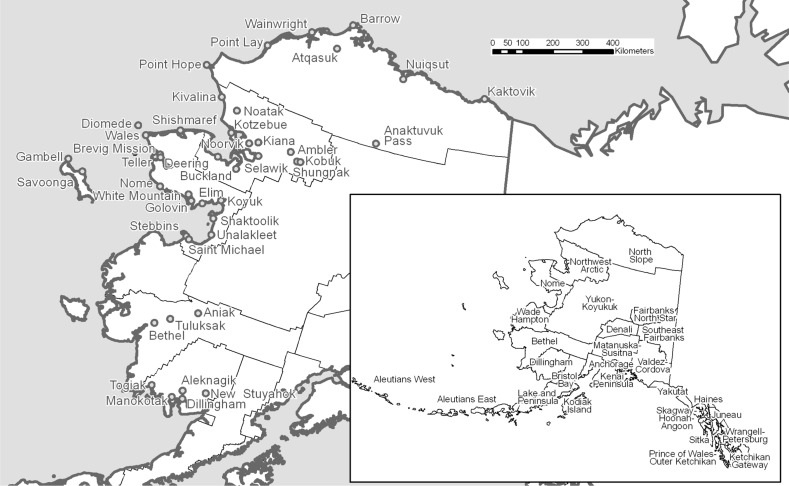
Forty-three selected Arctic Alaska towns and villages (*larger map*), representing five different boroughs or census areas (*inset*). From Hamilton and Mitiguy (2009)

Communities in Arctic Alaska are small, ranging in population from as few as 20 to over 6000. These communities are not on a state-wide road system and are accessible from the state’s urban centers mainly by air or barge. Within geographically large boroughs or census areas, sparse populations are typically concentrated in one regional hub town and a number of smaller villages. In this paper, we focus on a demographic database that covers 43 communities (Fig. 1) including five regional hubs—Barrow, Bethel, Dillingham, Kotzebue, and Nome—having 2014 populations of 2400–6200. Each of these five serves as the transportation, economic, and administrative center for its respective region. The remaining 38 places are smaller villages, having populations from about 100–900. Socioeconomic and infrastructure contrasts between hub towns and villages are greater, in some respects, than the contrasts between towns or between villages of different regions (Hamilton and Seyfrit 1993).

A majority of the population in both towns and villages is Alaska Native, mainly Inupiat or Yupik. In villages, their fraction often exceeds 85 %. With the overlay of mainstream and traditional societies, and mixed cash/subsistence economies, these remote communities face rapid change and complex problems (BurnSilver et al. 2016; Chance 1990; Fienup-Riordan 1990; Hamilton and Seyfrit 1994a, b; Loring et al. 2016; NRC 2003; Jorgensen 1990; Seyfrit et al. 1998). The challenges are not unique to Alaska but occur with variations among indigenous peoples in many parts of the circumpolar North (Andrew 2014; Einarsson et al. 2004; Glomsrød and Aslaken 2009; Hamilton and Rasmussen 2010; Hamilton et al. 2010; Huskey and Southcott 2010; Krupnik and Jolly 2002; Kruse et al. 2011; Larsen et al. 2010, 2015; Larsen and Fondahl 2015; Loring and Gerlach 2015; NRC 2003; Rasmussen et al. 2015; Young and Bjerregaard 2008).

Kivalina, an Inupiat village of some 400 people, is about 130 kilometers north of the Arctic Circle in northwest Alaska. It sits on a gravel bar between Kivalina Lagoon and the Chukchi Sea. With Arctic warming, sea ice has been forming later in the fall, leaving the low shoreline exposed to erosion from powerful storms. Tens of meters of ground have been lost already; barrier construction temporarily slowed the loss, but the US Army Corps of Engineers estimates the location will be uninhabitable by 2025. Evacuation from this narrow strip of land would be difficult in rough conditions, so a major storm could pose risks. In 2008, Kivalina filed suit against Exxon Mobil and 23 other fuel or power corporations, arguing that global warming threatened the village’s existence, and estimating that their relocation costs will reach $400 million (Faris 2008). Although a federal court dismissed the suit, Kivalina’s chronic predicament continues to draw journalistic attention with headlines such as “Will these Alaska villagers be America’s first climate change refugees?” (Wernick 2015; other examples include Associated Press 2013; La Ganga 2015). Barack Obama became the first president to visit the US Arctic in summer 2015, when he flew into the town of Kotzebue and viewed Kivalina from the air. The President used his visit to highlight impacts of climate change on Alaska communities, with Kivalina as a case in point (CBC 2015).

Although Kivalina’s predicament is stark, other communities face serious problems as well. Thawing permafrost weakens ground beneath the island community of Shishmaref (pop. 600), while sea ice decline leaves it exposed to wave erosion. As the shoreline retreats by more than three meters per year, houses have fallen into the sea, becoming iconic photo-images for climate change threats. Shaktoolik (pop. 260) and some others are in similarly precarious coastal locations, but not all of the climate-threatened communities are coastal. Selawik (pop. 850), for instance, is experiencing widespread building and infrastructure damage as ground subsides with thawing permafrost, while erosion consumes land along the banks of Selawik River and Selawik Lake. Throughout Arctic Alaska, less visible but dangerous impacts also come from shorter cold seasons and lower predictability for travel across ice.

Responding to statewide concern about climate change impacts, in 2007 Alaska’s governor established a Climate Change Sub-Cabinet. The Immediate Action Workgroup (IAWG), part of this cabinet, assessed risks to communities based on four criteria: (1) safety of life during a reasonably foreseeable storm or flood event; (2) potential loss of critical infrastructure; (3) health threats to the community as defined by CDC or the Health Department; and (4) potential loss of 10 % or more of residential dwellings (IAWG 2009). The IAWG analysis identified six “priority at-risk” communities facing the most urgent threats. Kivalina is of course on this list, along with Koyukuk, Newtok, Shaktoolik, Shishmaref, and Unalakleet (IAWG 2009).

Independently, the United States Army Corps of Engineers (USACE) conducted a baseline erosion assessment from 2005 to 2009 (USACE 2009). They applied a weighted scoring system based on: (1) critical infrastructure; (2) human health and safety; (3) subsistence and shoreline use; (4) community setting/geographic location; (5) housing and population affected; (6) housing in parallel; (7) environmental hazard; (8) cultural importance; and (9) commercial/non-residential. Scores on each criterion were summed for 178 communities; 26 of these (including Kivalina) having overall scores more than one standard deviation above the mean were characterized as “priority action” communities.

A third list of communities facing climate-related risks to their water infrastructure or resources was compiled Tetra Tech (2010), in a study by commissioned by the state government. The Tetra Tech assessment excluded places already identified as “priority at-risk” in the IAWG study. Their report lists 25 communities where water supplies are imperiled and need “priority study,” and 24 others also imperiled but in the less urgent category of concern, needing additional study.

These three reports provide alternative lists of which Alaska communities are most impacted or threatened by climate-related problems. Net out-migration is substantial from many parts of rural Alaska, but is it any greater from threatened places than elsewhere? If so, that could be a clear signal of climate impacts on migration, occurring even without government support. A demographic database covering 43 Arctic Alaska towns and villages, with yearly net migration estimates for 1991 through 2014, permits direct tests of this hypothesis.

## Data and methods

We focus here on a diverse set of communities from five Alaska regions: the North Slope and Northwest Arctic boroughs, along with the Nome, Bethel, and Dillingham census areas. Not all of these places lie north of the Arctic Circle, but they are characterized by generally Arctic landscapes and conditions. Within the five regions, 43 communities represent a range of environments and access to resources (Hamilton and Mitiguy 2009; Hamilton et al. 2012). Figure 1 maps these communities, with regions named in the inset.

Our database on these 43 places, organized with one observation for each place/year, includes temperature and precipitation estimates at each community’s location for every month since January 1979, derived from reanalysis described by Rienecker et al. (2011).^1^ Demographic variables include population, births, deaths, and net migration estimates for each community in each fiscal year (roughly July 1–June 30) since 1990. At this writing, the time series are complete through 2014.

Annual estimates of population in each community, between the decennial US Census years, come from the Alaska Department of Labor and Workforce Development (ADL 2015). These are based partly on Permanent Fund Dividend applications—a unique Alaska data resource that supports relatively accurate population estimates. Separately, the Alaska Bureau of Vital Statistics records births and deaths for each community. By combining population with births and deaths, we estimate the annual net migration.

**Fig. 2:**
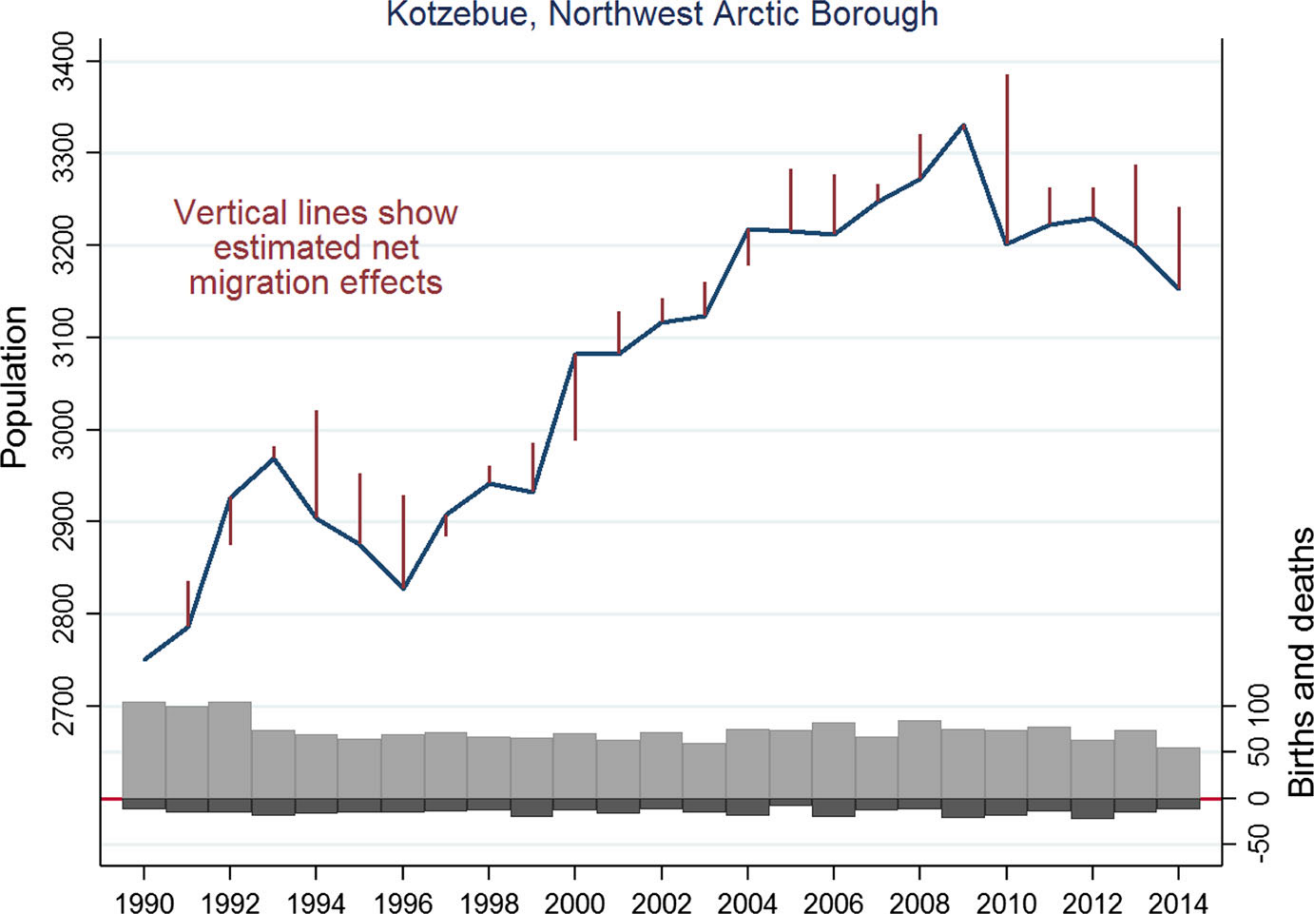
Population dynamics of Kotzebue, Alaska, 1990–2014. *Gray bars* show number of births and deaths; *vertical lines* indicate estimated net migration (population and birth/death numbers graphed from different baselines, but with comparable *y*-axis scales)

Time plots visualize population trends in each community, as illustrated for the Northwest Arctic hub town of Kotzebue in Fig. 2.^2^ Kotzebue’s 2014 population of about 3200 people is more than 70 % Alaska Native, primarily Inupiat. Bars in the lower portion of Fig. 2 indicate the total number of deaths (dark bars) and births (lighter bars) for each fiscal year, July 1–June 30 (original data from ADL 2015 and the Alaska Bureau of Vital Statistics; for definitions, see Saito et al. 2015). The number of deaths per year ranged from 9 to 23, and the number of births to residents from 55 to 105. These seemingly exact counts of births, deaths, and population are of course subject to minor errors. A scale for deaths and births appears at lower right in Fig. 2. On average, about 58 more births than deaths occurred each year. Without out-migration, the population would be constantly increasing.

Short line segments that extend above the main curve in Fig. 2 indicate net out-migration, inferred from a population that is lower than would be expected due to natural increase alone. For example, Kotzebue’s estimated population for July 1, 2013 was 3199. Over the next 12 months, 55 births and 12 deaths were recorded, so by natural increase alone the 2014 population should have been 3199 + 55 − 12 = 3242. This value is indicated by the top of the vertical line segment for 2014. In fact, however, the estimated 2014 population was 3153, suggesting net out-migration of 3242 − 3153 = 89 people—the length of the vertical line segment descending to the curve marking actual (estimated) population. A line segment below the main curve, as in 2000 or 2004, indicates net in-migration, or population growth exceeding that expected from natural increase. Although Kotzebue experienced a few years with net in-migration, in most years more people left than arrived. The average annual loss was about 40 people, partially offsetting natural increase, so that total population growth slowed and sometimes declined.

Diverse patterns of population change occur among the 43 Arctic communities. Different settlements within the same region can be growing, shrinking, or roughly stable, depending on net migration. Annual changes in small communities could reflect chance or unique local events. The next section steps back to view larger patterns.

## General patterns of change

In 1990, the 43 Arctic settlements had a combined population of about 29,000. By 2014, this had grown to almost 36,000. There were approximately 15,000 more births than deaths over this period, so natural increase was offset by net out-migration of nearly 8000 people. Year-to-year fluctuations are erratic in each place, but overall summaries help to clarify the big picture.

**Fig. 3:**
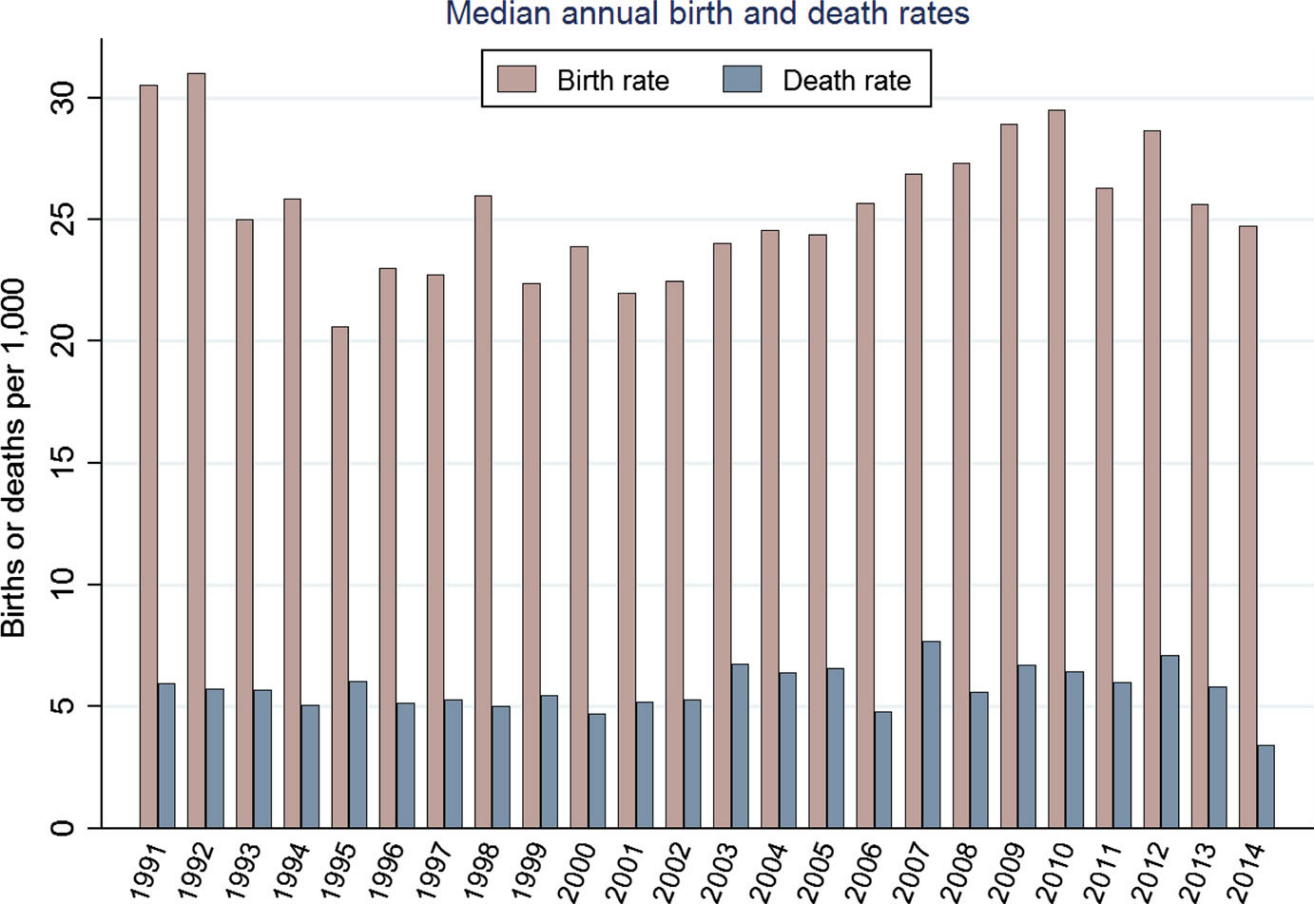
Median annual birth and death rates for 43 towns and villages, 1991–2014

Figure 3 graphs median annual crude birth and death rates for these communities from 1991 to 2014. (Medians and related tests are employed throughout our analysis, due to their resistance to outliers and better performance with skewed distributions.) The wide surplus of births over deaths in all years creates pressure toward continual population growth. Birth rates declined from initially high levels in the early 1990s and then gradually rose again after the mid-2000s, reflecting an echo boom from the earlier era of high birth rates. The final years hint at recent decline, but neither death nor birth rates exhibit sustained trends.

**Fig. 4:**
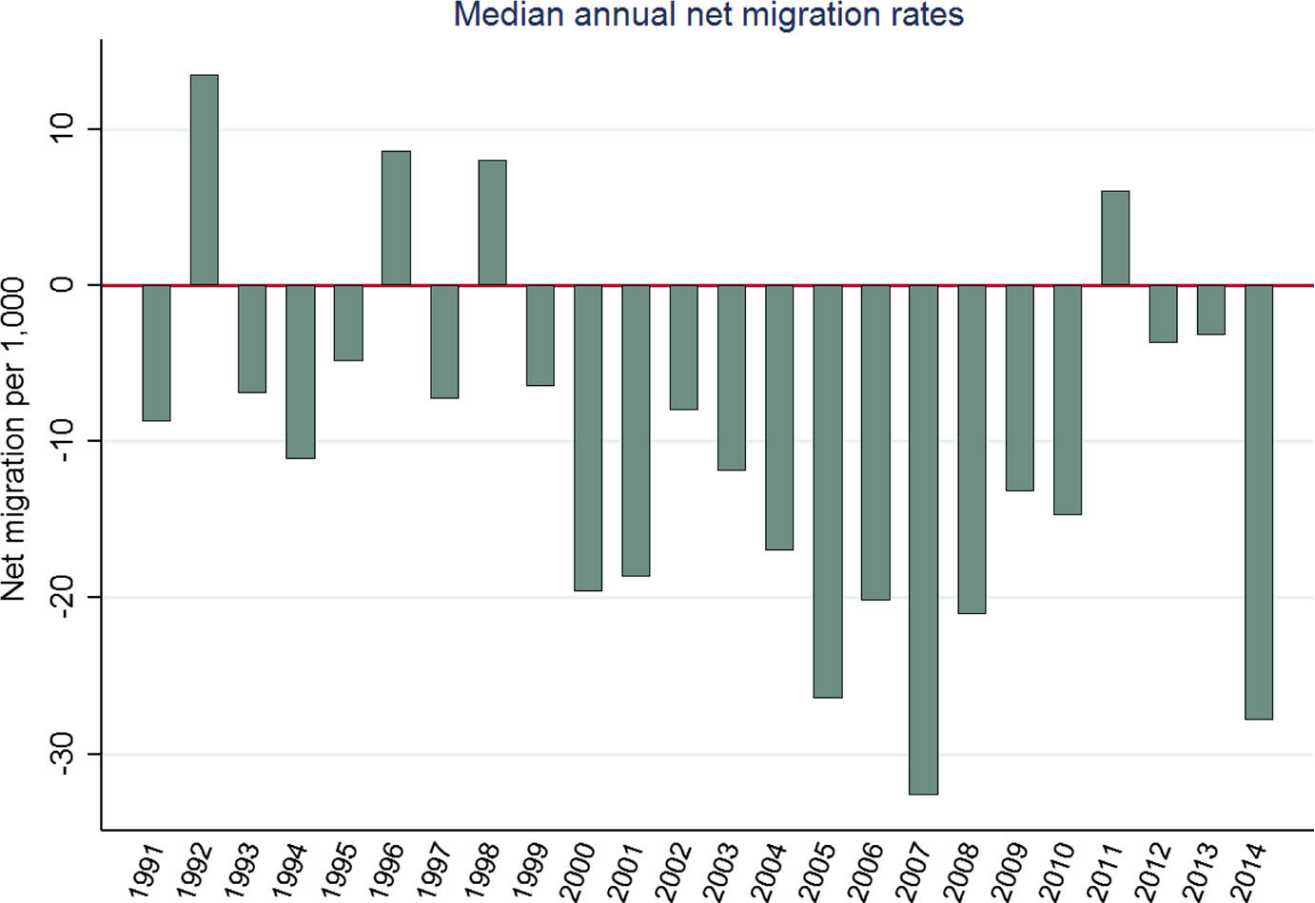
Median annual net migration rates for 43 towns and villages, 1991–2014

Figure 4 graphs the median net migration estimates for these 43 places over 1991–2014. Whereas natural increase was always positive, net migration for most years was negative. Sharp interannual swings invite speculation, but general explanations thus far have proven to be elusive. Net migration is often viewed as a social indicator in the North, sensitive to shifting push and pull factors (Hamilton 2010; Howe et al. 2014). Rapid response has been documented in connection with fisheries troubles in subarctic Alaska (Himes-Cornell and Hoelting 2015), Newfoundland (Hamilton and Butler 2001; Hamilton 2007) and the Faroe Islands (Hamilton et al. 2004), and in connection with economic and administrative contraction in post-Soviet Russia (Heleniak and Bogoyavlensky 2015; Voinov et al. 2004).

Caught between necessity, limited resources, and very limited supply options, rural Alaska communities are exceptionally vulnerable to the price of fuel and market foods (Ford et al. 2006; Loring and Gerlach 2009). Two events in 2008—a sharp rise in fuel prices early that year, and a global recession starting in August—seemed likely to have particularly harsh consequences that could drive out-migration (Martin et al. 2008). Counterintuitively, however, net out-migration in 2008 was less than the year before. Over the next 5 years, it went even lower. The lack of a migration response after 2008 suggests that these Alaska places exhibit less short-term sensitivity to economic events, compared with some other Northern places such as fisheries-dependent communities of the North Atlantic Arc (e.g., Hamilton and Butler 2001; Hamilton et al. 2004; Hamilton 2007). In the next section, we test for a longer-term response to climate stress.

## Migration from climate-stressed places

**Fig. 5:**
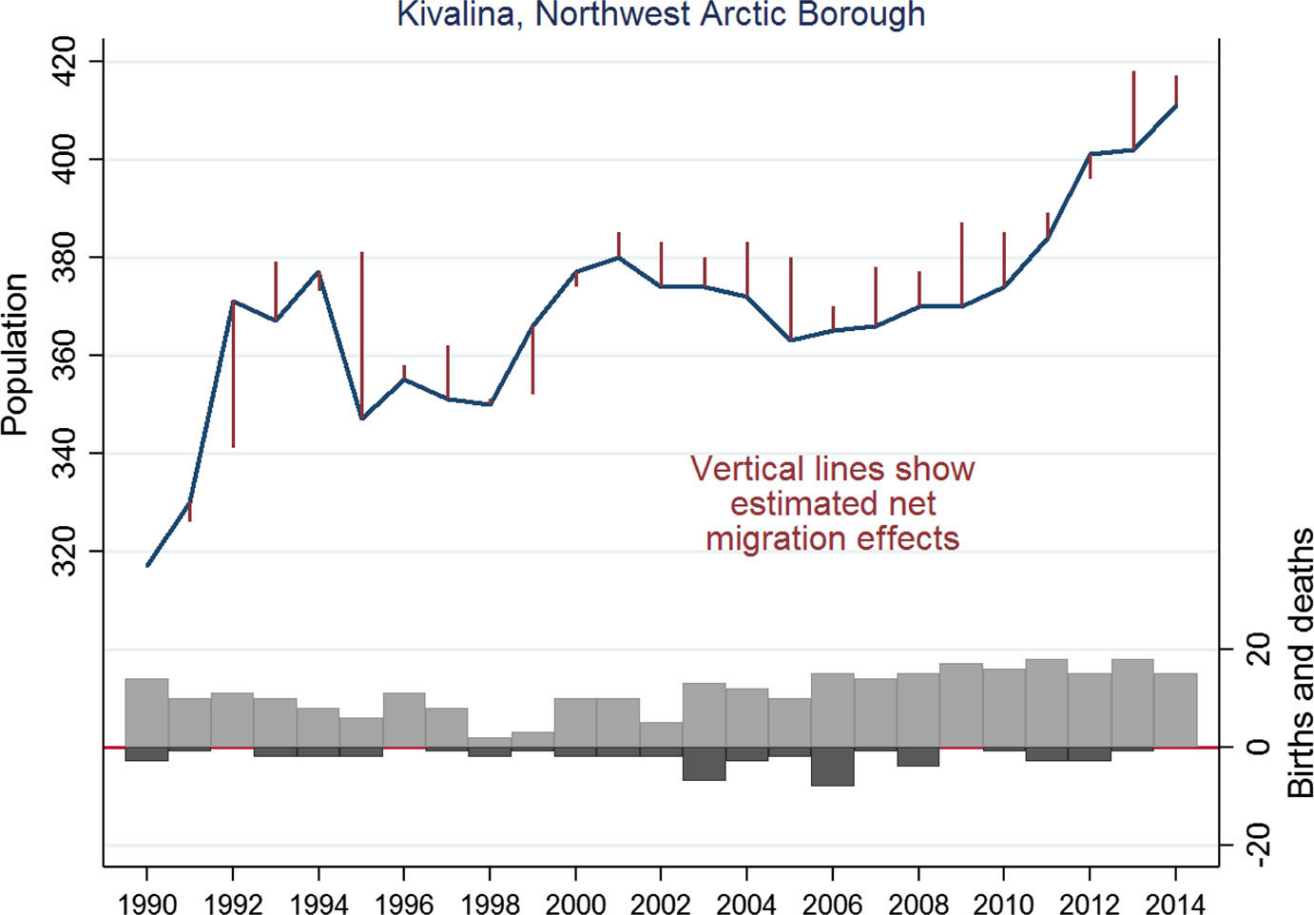
Population dynamics of Kivalina, Alaska, 1990–2014

The Northwest Arctic Borough village of Kivalina faces perhaps the most acute climate-related threats, both from inexorable erosion and the sudden danger of storms. The risks are known throughout the state and all too obvious to Kivalina residents, who have seen ground lost a few steps from homes and school, and experienced several storm-driven evacuations (Lane 2012). Federal and state efforts to date have not approached levels needed to move people out permanently or relocate the whole village, nor are residents of one mind on what should happen. But while top-level decisions are not made, might some individuals and families vote with their feet? Kivalina’s population history, graphed in Fig. 5, gives no indication of this happening on a substantial scale. Out-migration has been consistent but relatively small, so the population has continued to grow—up by almost 50 people (a 12 % increase) since 2005.

What about other places? Our demographic database includes many of those listed as threatened in three reports described earlier. Four database communities, including Kivalina, are classed as “priority at-risk” in IAWG (2009). Ten database communities (including these four) are classed as “priority action” in the USACE (2009) report. The Tetra Tech report (2010) provides a third, non-overlapping list of places warranting “priority” or “concern.” Eighteen of these places also occur in our data. These three reports thus provide alternative ways to classify subsets of Arctic Alaska communities that are most stressed by climate change-related erosion and water problems.

Table 1 makes a systematic comparison of net migration and population change rates, contrasting USACE, IAWG, and Tetra Tech prioritized places with the others in our data. To focus on recent developments as erosion and water problems became more acute, only the years from 2000 to 2014 are used for this analysis (43 places × 15 years = 645 place/years). Medians and confidence intervals for these groups over 2000 to 2014 are estimated by quantile regression, as are the *F* tests for differences among medians (more precisely, differences among 0.5 quantiles). The quantile approach does not require normal or Gaussian errors, which are implausible for these data. Given the clustered data structure, we also cannot assume that errors are independent and identically distributed. Consequently, the analysis employs robust standard errors or variance estimates for all confidence intervals and tests (Koenker 2005; calculations done with Stata 14.1, see Hamilton 2013).

**Table 1:** Median annual 2000–2014 rates of net migration and population change, shown with 95 % confidence intervals and *F* tests from quantile regression using robust standard errors, comparing erosion-threatened, and other Arctic Alaska communities (*n* = 645 place/years)

Classification (#)	Median annual	Median annual
	Net migration %	Population change %
*USACE* 2009		
Priority action (10)	−1.50 (−2.04, −0.96)	0.65 (0.17, 1.14)
Others (33)	−1.60 (−2.04, −1.17)	0.31 (−0.14, 0.77)
*F* test	*F*(1, 643) = 0.08, *p* = 0.77	*F*(1, 643) = 1.00, *p* = 0.32
*IAWG* 2009		
Priority at-risk (4)	−1.43 (−2.04, −0.82)	0.72 (0.18, 1.25)
Others (39)	−1.65 (−2.04, −1.27)	0.38 (−0.00, 0.77)
*F* test	*F*(1, 643) = 0.36, *p* = 0.55	*F*(1, 643) = 0.97, *p* = 0.33
*Tetra Tech* 2010		
Priority study (11)	−1.76 (−2.67, −0.86)	0.70 (−0.23, 1.61)
Concern (7)	−2.37 (−3.69, −1.06)	0.00 (−1.23, 1.23)
Others (25)	−1.46 (−1.84, −1.08)	0.44 (0.08, 0.79)
*F* test	*F*(2, 642) = 0.97, *p* = 0.38	*F*(2, 642) = 0.40, *p* = 0.67
**All (43)**	−1.57 (−1.93, −1.21)	0.44 (0.09, 0.78)

Regardless of which classification scheme we use, none of the comparisons shows significant differences in median net migration or population change rates—as seen either from *F* tests or the overlapping confidence intervals in Table 1. Thus, we have no evidence of greater net out-migration from the priority communities, or of lower rates of population change. If anything, the threatened places (by either USACE or IAWG criteria) are growing faster than others: Their median net migration is slightly less negative, and population growth rates slightly higher.

**Fig. 6:**
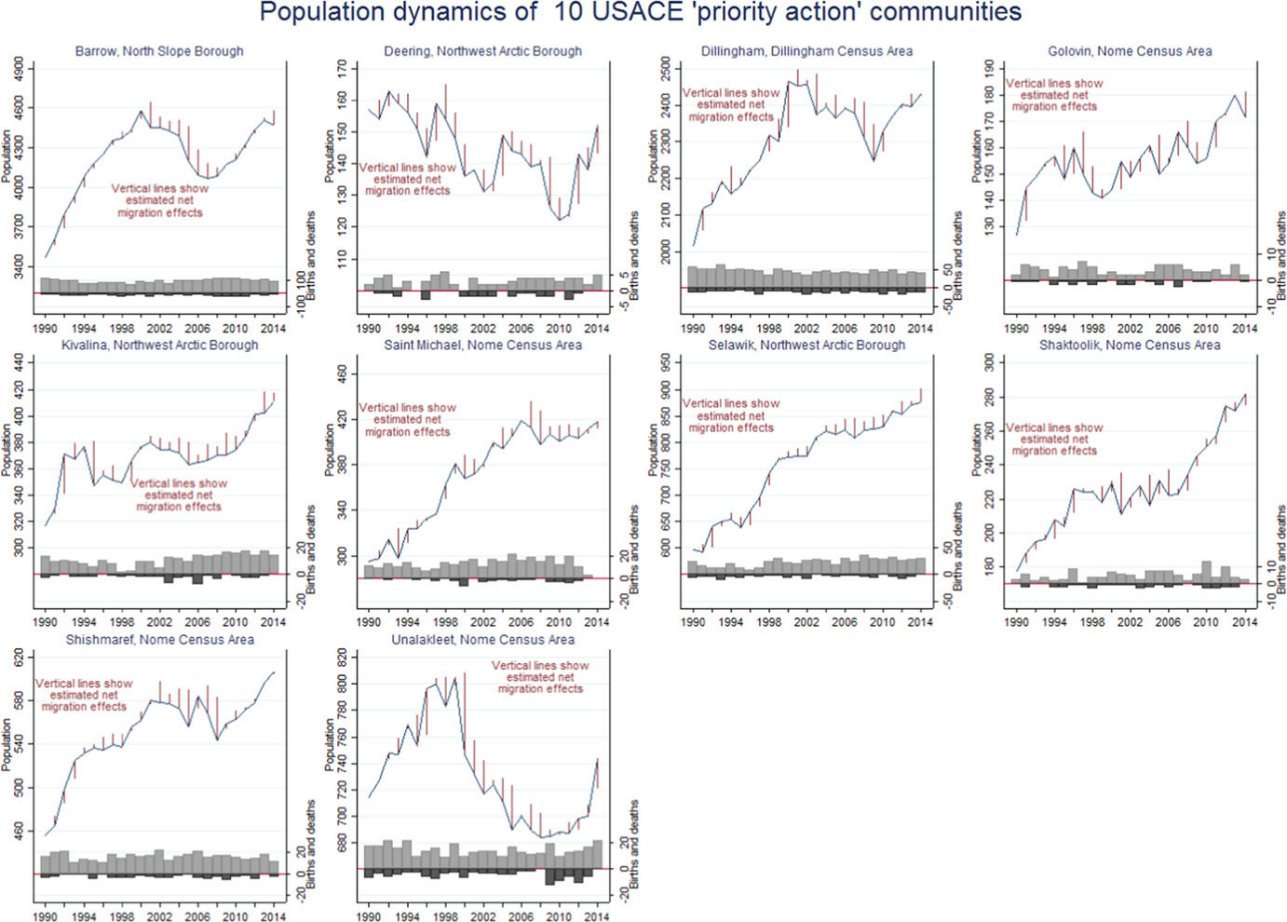
Population dynamics of 10 “priority action” communities identified by the US Army Corps of Engineers (USACE 2009). Kivalina, Shaktoolik, Shishmaref, and Unalakleet are also among the “priority at-risk” communities identified by IAWG (2009)

Figure 6 elaborates the summary results graphically by showing 1990–2014 population change in the 10 USACE “priority action” communities that occur in our Arctic dataset. (The four IAWG “priority at-risk” communities in our data—Kivalina, Shaktoolik, Shismaref, and Unalakleet—comprise a subset of these 10, so they also appear in Fig. 6.) These 10 places exhibit a range of different patterns. For example, Selawik grew steadily with minor out-migration through these years. With proportionately more out-migration, the population of Deering gradually declined. Several places show signs of growth pausing or reversing due to higher net out-migration in the middle of this period, but the timing is not synchronous, and slow or negative growth has been followed by more recent increase. Unalakleet stands out visually in Fig. 6 due to its relatively large decline (more than 100 people) after 1999, corresponding to collapse and closure of the local king salmon fishery (Kent and Bergstrom 2012). Even in Unalakleet, however, population subsequently resumed growing.

Both the formal tests in Table 1 and the informal comparison in Fig. 6 underscore the lack of evidence for climigration to date. People are not leaving at higher rates; on the contrary, rising populations in many places mean that more people are potentially exposed to adverse effects now than when these reports were commissioned or published their warnings.

## Discussion

In the original usage of *climigration* (Bronen 2009), and discussions about the threatened Alaska communities, climate-driven relocation has been framed largely in terms of the need for government action. But government action has been limited (Marino 2015) and given the rising costs of relocation that may not soon be forthcoming. Outside Alaska, climate-driven migration is often framed in terms of individual or family agency, occurring with or without government support. Many factors enter into migration decisions, however, making it difficult to distinguish a climatic component. Our quasi-experimental approach involves tracking net migration over time in threatened and non-threatened communities, and comparing overall migration rates of otherwise similar places classified by risk criteria.

The hypothesis of unplanned climigration seems plausible for Arctic Alaska communities because in recent history, their migration flows have been volatile, substantially affecting populations (Hamilton and Mitiguy 2009). Surveys of Arctic Alaska high school students in the 1990s found that more than half expected to migrate permanently away from their home regions (Hamilton and Seyfrit 1993; Seyfrit et al. 1998). Although actual out-migration rates prove to be lower, with many non-permanent moves, going away (or not) remains a major life choice that measurably affects source community demographics—for example, by skewing sex ratios as women disproportionately leave the villages (Hamilton and Seyfrit 1994b; Howe 2009; Huskey et al. 2004; Martin 2009; for a European counterpart, see Leibert 2015). Individual and family migration decisions have similarly large effects in many other parts of the North (Hamilton et al. 2004; Hamilton and Rasmussen 2010; Heleniak and Bogoyavlensky 2015; Himes-Cornell and Hoelting 2015; Huskey and Southcott 2010; Larsen et al. 2010, 2015; Rasmussen 2011).

Migration is a complex phenomenon. Black et al. (2011) suggest a non-regional framework for understanding how the environment, acting together with economic, political, social, and demographic drivers, affects migration. Direct environmental pressures include impacts on ecosystem services, which in the Alaska case support subsistence food harvesting and inter-community travel, along with settlement infrastructure including homes, schools, water, and power supplies. Moreover, environmental change can raise what Black et al. term “the hazardousness of place,” a description that well suits Alaska communities with growing flood risks to infrastructure and people. Black et al. emphasize the role of human agency in migration decisions, a role that underlies our use of net migration as an indicator. But this indicator shows no visible response to environmental stress to date, which serves to emphasize that human agency can work against migration as well as for it. Pressures to move are countered by reasons people might not want to leave their ancestral homes, or lack appealing alternatives (Adams 2015; Marino 2015). La Ganga (2015) quotes a longtime Kivalina resident:

When… asked why her people don’t move—somewhere, anywhere to be safe—she is polite but firm. The land and the water make the Inupiat who they are. If they moved to Kotzebue, they would be visitors. Moving to Anchorage or Fairbanks, she said, “would be like asking us not to be a people any more.”

Instead of their populations declining through out-migration, many of the threatened places are growing—raising both the potential costs of relocation and the number of people exposed to risks. Meanwhile, the physical threats are not diminished. Permafrost thaws, rivers rise, and shorelines erode; on decadal timescales, sea level could be an increasing factor as well. State or federal action sufficient to relocate these communities does not yet appear imminent.

The fact that imperiled rural communities are growing despite net out-migration raises a final question about how demographics are changing in the state, and how they will influence vulnerability to climate change and extreme events. Some limited work has been done looking at how specific demographic groups—women, men, and the elderly, for example—are differentially impacted by climatic and environmental change (Beaumier and Ford 2010; Graves 2005; Lewis 2011), but generally, much more needs to be known about who is staying in these communities, why, and what these changes mean in terms of the likely human burden of climate change and extreme events moving forward.

To be clear, our analysis does not undermine the original point of Alaska climigration discussions: the need for government-supported relocation. Nor does it cast any doubt on the urgency of this problem, especially in places where climate-linked erosion poses a growing threat to infrastructure, homes, and safety. If anything, as these places continue to grow and change demographically, our analysis suggests that the urgency of problem is increasing. Relocation or individual movements will certainly occur from the most exposed locations, and hopefully before disasters occur. The scope of future climate-related migration should be visible through analysis along the lines of this paper, and the general approach could be applied elsewhere as well. Initially conceived as one social science counterpart to the Arctic monitoring efforts of natural scientists (AMAP 2016; SAON 2016), the community-level time series follow in the spirit of the Arctic Observing Network (Kruse et al. 2011) and Arctic Social Indicators (Larsen et al. 2010, 2015) projects. Both social and physical data reveal an Arctic rapidly changing.

## Footnotes

1 The complete database is published online (Saito et al. 2015).

2 A full set of 43 population plots, along with others showing time series of temperature and precipitation at each community's location, appear in Saito et al. 2015 and can be freely used with acknowledgment.

## References

[CR1] Adams H (2015). Why populations persist: Mobility, place attachment and climate change. Population and Environment.

[CR2] ADL (Alaska Department of Labor and Workforce Development). (2015). *Population estimates*. http://laborstats.alaska.gov/pop/popest.htm. Accessed September 2, 2015.

[CR3] AMAP. (2016). Arctic monitoring and assessment program. http://www.amap.no/.

[CR4] Andrew, R. (2014). *Socio*-*economic drivers of change in the arctic*. AMAP technical report no. 9. Oslo: Arctic Monitoring and Assessment Program. http://www.amap.no/documents/doc/Socio-Economic-Drivers-of-Change-in-the-Arctic/1115. Accessed November 11, 2015.

[CR5] Associated Press. (2013). Alaska seeks federal money to move a village threatened by climate change. October 3. http://www.nytimes.com/2015/10/04/us/alaska-seeks-federal-money-to-move-a-village-threatened-by-climate-change.html?_r=0.

[CR6] Beaumier M, Ford JD (2010). Food insecurity among Inuit women exacerbated by socio-economic stresses and climate change. Canadian Journal of Public Health.

[CR7] Black R, Adger WN, Arnell NW, Dercon S, Geddes A, Thomas D (2011). The effect of environmental change on human migration. Global Environmental Change.

[CR8] Bronen R, Oliver-Smith A, Shen X (2009). Forced migration of Alaskan indigenous communities due to climate change: Creating a human rights response. Linking environmental change, migration and social vulnerability.

[CR9] Bronen R, Chapin FS (2013). Adaptive governance and institutional strategies for climate-induced community relocations in Alaska. Proceedings of the National Academy of Sciences.

[CR10] BurnSilver S, Magdanz J, Stotts R, Berman M, Kofinas G (2016). Are mixed economies persistent or transitional? Evidence using social networks from Arctic Alaska. American Anthropologist.

[CR11] CBC (Canadian Broadcasting Corporation). (2015). *Barack Obama gives hope to Alaskan village affected by climate change. September 10*. http://www.cbc.ca/news/canada/north/barack-obama-gives-hope-to-alaskan-village-affected-by-climate-change-1.3221857. Accessed December, 23, 2015.

[CR12] Chance NA (1990). The inupiat and Arctic Alaska: An ethnography of development.

[CR13] Corbett M (2005). Rural education and out-migration: The case of a coastal community. Canadian Journal of Education.

[CR14] Corbett MJ (2007). Learning to leave: The irony of schooling in a coastal community.

[CR15] Curtis K, Schneider A (2011). Understanding the demographic implications of climate change: Estimates of localized population predictions under future scenarios of sea-level rise. Population and Environment.

[CR16] DeWaard J, Curtis KJ, Fussell E (2016). Population recovery in New Orleans after Hurricane Katrina: Exploring the potential role of stage migration in migration systems. Population and Environment.

[CR17] Einarsson N, Larsen JN, Nilsson A, Young OR (2004). Arctic human development report.

[CR18] Entwisle BNE, Williams AM, Verdery RR, Rindfuss SJ, Walsh GP, Malanson PJ, Mucha BG, Frizzelle PM, McDaniel X, Yao BW, Heumann P, Prasartkul Y Sawangdee, Jampaklay A (2016). Climate shocks and migration: An agent-based modeling approach. Population and Environment.

[CR19] Faris S (2008). Conspiracy theory. The Atlantic.

[CR20] Field CB, Barros VR, Dokken DJ, Mach KJ, Mastrandrea MD, Bilir TE, Chatterjee M, Ebi KL, Estrada YO, Genova RC, Girma B, Kissel ES, Levy AN, MacCracken S, Mastrandrea PR, White LL (2014). Climate change 2014: Impacts, adaptation, and vulnerability. Part A: Global and sectoral aspects. Contribution of working group II to the fifth assessment report of the intergovernmental panel on climate change.

[CR21] Fienup-Riordan A (1990). Eskimo essays: Yup’ik lives and how we see them.

[CR22] Finch C, Emrich CT, Cutter SL (2010). Disaster disparities and differential recovery in New Orleans. Population and Environment.

[CR23] Ford JD, Smit B, Wandel J (2006). Vulnerability to climate change in the Arctic: A case study from Arctic Bay, Canada. Global Environmental Change.

[CR24] GAO (US General Accounting Office). (2009). *Alaska native villages: Limited progress has been made on relocating villages threatened by flooding and erosion*. Government Accountability Office Report to Congressional Requesters. http:www.gao.gov/new.items/d09551.pdf. Accessed September 19, 2015.

[CR25] Gerlach SC, Loring PA, Turner AM, Atkinson DE, Lovecraft AL, Eicken H (2011). “Food systems, climate change, and community needs. North by 2020.

[CR26] Glomsrød, S., & Aslaken, I (eds.) (2009). *The economy of the North 2008*. Oslo: Statistics Norway. https://oaarchive.arctic-council.org/handle/11374/35. Accessed November 11, 2015.

[CR27] Graves, K. (2005). *Resilience and adaptation among Alaska Native men*. M.A. Thesis, Department of Psychology. Anchorage, AK: University of Alaska.

[CR28] Hamilton LC (2007). Climate, fishery and society interactions: Observations from the North Atlantic. Deep Sea Research II.

[CR29] Hamilton LC, Huskey L, Southcott C (2010). Footprints: Demographic effects of out-migration. Migration in the Circumpolar North: Issues and contexts.

[CR30] Hamilton LC (2013). Statistics with Stata, version 12.

[CR31] Hamilton LC, Butler MJ (2001). Outport adaptations: Social indicators through Newfoundland’s cod crisis. Human Ecology Review.

[CR32] Hamilton LC, Mitiguy AM (2009). Visualizing population dynamics of Alaska’s Arctic communities. Arctic.

[CR33] Hamilton LC, Rasmussen RO (2010). Population, sex ratios and development in Greenland. Arctic.

[CR34] Hamilton LC, Seyfrit CL (1993). Town–village contrasts in Alaskan youth aspirations. Arctic.

[CR35] Hamilton LC, Seyfrit CL (1994). Female flight? Gender balance and out-migration by Native Alaskan villagers. Arctic Medical Research.

[CR36] Hamilton LC, Seyfrit CL (1994). Coming out of the country: Community size and gender balance among Alaskan Natives. Arctic Anthropology.

[CR37] Hamilton LC, Colocousis CR, Johansen STF (2004). Migration from resource depletion: The case of the Faroe Islands. Society and Natural Resources.

[CR38] Hamilton LC, Bjerregaard P, Poppel B, Larsen JN, Schweitzer P, Fondahl G (2010). Health and population. Arctic social indicators.

[CR39] Hamilton LC, White DM, Lammers RB, Myerchin G (2012). Population, climate and electricity use in the Arctic: Integrated analysis of Alaska community data. Population and Environment.

[CR40] Heleniak T, Bogoyavlensky D, Larsen JN, Fondahl G (2015). Arctic populations and migration. Arctic human development report: Regional processes and global linkages.

[CR41] Henry S, Schoumaker B, Beauchemin C (2004). The impact of rainfall on the first out-migration: A multi-level event-history analysis in Burkina Faso. Population and Environment.

[CR42] Himes-Cornell A, Hoelting K (2015). Resilience strategies in the face of short- and long-term change: Out-migration and fisheries regulation in Alaskan fishing communities. Ecology and Society.

[CR43] Howe EL (2009). Patterns of migration in Arctic Alaska. Polar Geography.

[CR44] Howe EL, Huskey L, Berman MD (2014). Migration in Arctic Alaska: Empirical evidence of the stepping stones hypothesis. Migration Studies.

[CR45] Hunter L (2005). Migration and environmental hazards. Population and Environment.

[CR46] Hunter LM, Luna JK, Norton RM (2015). Environmental dimensions of migration. Annual Review of Sociology.

[CR47] Huntington HP, Goodstein E, Euskirchen E (2012). Towards a tipping point in responding to change: Rising costs, fewer options for arctic and global societies. Ambio.

[CR48] Huskey L, Southcott C (2010). Migration in the Circumpolar North: Issues and context.

[CR49] Huskey L, Berman M, Hill A (2004). Leaving home, returning home: Migration as a labor market choice for Alaska Natives. Annals of Regional Science.

[CR50] Hutton D, Haque CE (2004). Human vulnerability, dislocation and resettlement: Adaptation processes of river-bank erosion-induced displacees in Bangladesh. Disasters.

[CR51] IAWG (Immediate Action Workgroup). (2009). *Recommendations report to the governor’s subcabinet on climate change*. http://climatechange.alaska.gov/docs/iaw_rpt_17apr08.pdf. Accessed 24 June 2014.

[CR52] Islam M, Hasan M (2015). Climate-induced human displacement: A case study of Cyclone Aila in the south-west coastal region of Bangladesh. Natural Hazards.

[CR53] Jorgensen JG (1990). Oil age Eskimos.

[CR54] Kent, S. M., & Bergstrom, D. J. (2012). *Norton sound subdistrict 5 (Shaktoolik) and Subdistrict 6 (Unalakleet) King Salmon Stock Status and Action Plan, 2013*. Anchorage, AK: Alaska Department of Fish and Game. http://www.adfg.alaska.gov/FedAidpdfs/SP12-28. Accessed January 1, 2015.

[CR55] Koenker R (2005). Quantile regression.

[CR56] Krupnik I, Jolly D (2002). The earth is faster now: Indigenous observations of arctic environmental change.

[CR57] Kruse J, Lowe M, Haley S, Hamilton L, Berman M (2011). Arctic observing network social indicators project overview. Polar Geography.

[CR58] La Ganga, M. (2015). This is climate change: Alaska villagers struggle as island is chewed up by the sea. *Los Angeles Times* August 30. http://www.latimes.com/nation/la-na-arctic-obama-20150830-story.html. Accessed September 2, 2015.

[CR59] Lane, M. (2012). *Kivalina endangered: An eroding village*. School of Journalism, University of Alaska at Fairbanks. http://www.uafjournalism.com/extreme/index.php/frozen-phenomena/kivalina-weathers-the-storm-but-for-how-long. Accessed December 29, 2015.

[CR60] Larsen JN, Fondahl G (2015). Arctic human development report: Regional processes and global linkages.

[CR61] Larsen JN, Schweitzer P, Fondahl G (2010). Arctic social indicators.

[CR62] Larsen JN, Schweizer P, Petrov A (2015). Arctic social indicators II: Implementation.

[CR63] Leibert T (2015). She leaves, he stays? Sex-selective migration in rural East Germany. Journal of Rural Studies.

[CR64] Lewis JP (2011). Successful aging through the eyes of Alaska Native elders. What it means to be an elder in Bristol Bay, AK. The Gerontologist.

[CR65] Loebach P (2016). Household migration as a livelihood adaptation in response to a natural disaster: Nicaragua and Hurrican Mitch. Population and Environment.

[CR66] Loring PA, Gerlach SC (2009). Food, culture, and human health in Alaska: An integrative health approach to food security. Environmental Science & Policy.

[CR67] Loring PA, Gerlach SC (2015). Searching for progress on food security in the North American North: A Research synthesis and meta-analysis of the peer-review literature. Arctic.

[CR68] Loring PA, Gerlach SC, Penn H (2016). Community work in a climate of adaptation: Responding to change in rural Alaska. Human Ecology.

[CR69] Marino E (2012). The long history of environmental migration: Assessing vulnerability construction and obstacles to successful relocation in Shishmaref, Alaska. Global Environmental Change.

[CR70] Marino E (2015). Fierce climate, sacred ground.

[CR71] Marino E, Lazrus H (2015). Migration or forced displacement? The complex choices of climate change and disaster migrants in Shishmaref Alaska and Nanumea, Tuvalu. Human Organization.

[CR72] Martin S (2009). The effects of female out-migration on Alaska villages. Polar Geography.

[CR73] Martin, S., Killorin, M., & Colt, S. (2008). *Fuel costs, migration, and community viability*. Anchorage, AK: Institute of Social and Economic Research. https://scholarworks.alaska.edu/handle/11122/4429. Accessed January 1, 2016.

[CR74] Massey DS, Axinn WG, Ghimire DJ (2010). Environmental change and out-migration: Evidence from Nepal. Population and Environment.

[CR76] NOAA (National Oceanic and Atmospheric Administration). (2015). *Arctic report card: Update for 2015*. http://www.arctic.noaa.gov/reportcard/index.html. Accessed December 27, 2015.

[CR77] NRC (National Research Council). (2003). Cumulative environmental effects of oil and gas development on Alaska’s North Slope.

[CR78] Overpeck J, Sturm M, Francis JA, Perovich DK, Serreze MC, Benner R, Carmack EC, Chapin FS, Gerlach SC, Hamilton LC, Hinzman LD, Holland M, Huntington HP, Key JR, Lloyd AH, MacDonald GM, McFadden J, Noone D, Prowse TD, Schlosser P, Vörösmarty C (2005). Arctic system on trajectory to new state. EOS.

[CR79] Parenti C (2011). Tropic of chaos: Climate change and the new geography of violence.

[CR80] Rasmussen, R. O. (Ed.). (2011). *Megatrends*. Copenhagen: Nordic Council of Ministers. http://www.nordregio.se/en/Publications/Publications-2011/Megatrends/. Accessed September 21, 2015.

[CR81] Rasmussen RO, Roto J, Hamilton LC, Larsen JN, Schweizer P, Petrov A (2015). West-Nordic Region. Arctic social indicators II: Implementation.

[CR82] Rienecker MM, Suarez MJ, Gelaro R (2011). MERRA: NASA’s modern-era retrospective analysis for research and applications. Journal of Climate.

[CR100] Sabathil G (2010). A European vision for addressing global security threats. European View.

[CR83] Saito, K., Hamilton, L., Lammers, R., & Glidden, S. (2015). *Arctic Alaska: A reference library for 43 towns and villages*. UCAR/NCAR—CISL—ACADIS. Dataset. doi:10.5065/D6930R6J.

[CR84] SAON. (2016). *Sustaining arctic observing networks*. http://www.arcticobserving.org/. Accessed May 23, 2016.

[CR85] Seyfrit CL, Hamilton LC, Duncan CM, Grimes J (1998). Ethnic identity and aspirations among rural Alaska youth. Sociological Perspectives.

[CR86] Tetra Tech. (2010). *Imperiled community water resources analysi*s. Immediate Action Workgroup, An Advisory Group of the Governor’s Climate Change Sub-Cabinet. http://www.climatechange.alaska.gov/docs/iaw_tt_imperiled_h2o_30jun10.pdf. Accessed August 31, 2015.

[CR87] Tribal Climate Change Project. (2010). Climate change: Realities of relocation for Alaska Native villages. University of Oregon. http://tribalclimate.uoregon.edu/files/2010/11/AlaskaRelocation_04-13-11.pdf. Accessed December 23, 2015.

[CR88] USACE (United States Army Corps of Engineers). (2006). Relocation *Planning project master plan: Kivalina, Alaska*. http://www.poa.usace.army.mil/Portals/34/docs/civilworks/reports/KivalinaMasterPlanMainReportJune2006.pdf. Accessed September 28, 2015.

[CR89] USACE (United States Army Corps of Engineers). (2009). Alaska baseline erosion assessment: Study findings and technical report. http://www.poa.usace.army.mil/Portals/34/docs/civilworks/BEA/AlaskaBaselineErosionAssessmentBEAMainReport.pdf. Accessed June 26, 2014.

[CR90] Voinov A, Bromley L, Kirk E, Korchak A, Farley J, Moiseenko T, Krasovskaya T, Makarova Z, Megorski V, Selin V, Kharitonova G, Edson R (2004). Understanding human and ecosystem dynamics in the Kola Arctic: A participatory integrated study. Arctic.

[CR91] Wernick, A. (2015). Will these Alaska villagers be America’s first climate change refugees? *Public Radio International*. http://www.pri.org/stories/2015-08-09/will-residents-kivalina-alaska-be-first-climate-change-refugees-us. Accessed September 29, 2015.

[CR200] Young, T. K., & Bjerregaard, P. (Eds.). (2008). *Health transitions in Arctic populations*. Toronto: University of Toronto Press.

